# Point prevalence of non-melanoma and melanoma skin cancers in Australian surfers and swimmers in Southeast Queensland and Northern New South Wales

**DOI:** 10.7717/peerj.13243

**Published:** 2022-04-28

**Authors:** Mike Climstein, Brendan Doyle, Michael Stapelberg, Nedeljka Rosic, Isolde Hertess, James Furness, Vini Simas, Joe Walsh

**Affiliations:** 1 Aquatic Based Research/Faculty of Health, Southern Cross University, Bilinga, Queensland, Australia; 2 Exercise and Sport Science Exercise, Health & Performance Faculty Research Group Faculty of Health Sciences, University of Sydney, Sydney, NSW, Australia; 3 Water Based Research Unit, Bond University, Robina, Queensland, Australia; 4 John Flynn Specialist Centre, Advanced Skin Care, Tugan, Queensland, Australia; 5 Biomedical Science/Faculty of Health, Southern Cross University, Bilinga, Queensland, Australia; 6 Sports Science Institute, Sydney, NSW, Australia

**Keywords:** Physical activity, Questionnnaire, Skin neoplasm, Melanoma, Non-melanoma, Ultraviolet radiation, Surf, Swim

## Abstract

**Background:**

Surfing and swimming are two popular outdoor aquatic activities in Australia with an estimated 2.7 million surfers and three million swimmers; however, these activities are associated with intermittent exposure to ultraviolet radiation. Our aim was to determine the point prevalence of pre-skin cancer (actinic keratosis (PSC)), non-melanoma (NMSC) and melanoma skin cancers (MSC) in Australian surfers and swimmers.

**Methods:**

This cross-sectional study involved Australian surfers who completed a survey that included physiological demographics, aquatic activity-specific demographics, history of skin cancer followed by screening.

**Results:**

A total of 171 surfers (*n* = 116) and swimmers (*n* = 55) participated in the study. Both groups were identified as having a history of skin cancer (surfers 41.4%, swimmers 36.4%) and a family history of skin cancer (surfers 52.6%, swimmers 43.6%). The majority of both groups reported using a high percentage of a chemical or physical skin cancer prevention strategy (surfers 100%, Swimmers 92.7%, *P* = 0.003). Significantly more surfers were identified with a skin cancer of any type *vs*. swimmers (50% *vs*. 27.3%; OR 2.67; *P* = 0.005) with most the common skin cancer being PSC (44.7% *vs*. 11.3%, *P* = 0.076) followed by basal cell carcinoma (BCC) (24.2% *vs*. 7.6%, *P* = 0.068). There was a total of seven MSC identified in surfers and swimmers (4.6% *vs*. 0.8%, respectively, *P* = 0.137). Most skin cancers in surfers were located on the face (28.0%) followed by the arm and back (12.1% each), whereas in swimmers, the majority of skin cancers were identified on the face (17.3%), followed by the arm and lower leg (15.4% each). The highest number of melanomas were identified in surfers (*n* = 6) and mainly located on the face (*n* = 2) and back (*n* = 2). There was a single melanoma identified on the back in a swimmer. With the groups combined, the majority (42.9%) of melanomas were identified on the back in participants, followed by the face (28.6%). Rates per 100,000 of NMSC and MSC in surfers and swimmers (respectively) were BCC (11,206 *vs*. 14,545), squamous cell carcinoma (SCC) *in situ* (13,793 *vs*. 12,727), SCC (1,724 *vs*. 3,636) and MSC (5,172 *vs*. 1,818). When compared to the general Australian population, surfers and swimmers had higher odds ratios (OR), which included BCCs (OR 7.3 and 9.4, respectively), SCCs (OR 1.7 and 3.5, respectively) and MSC (OR 96.7 and 18.8, respectively).

**Conclusion:**

Surfers and swimmers had consistently higher rates of PSC, NMSC and MSC than the general Australian population. Point prevalence of MSC (groups combined) was 76-fold higher than the general Australian population. These findings highlight the clinical importance of regular skin cancer screenings in individuals who surf or swim for early detection and treatment of skin cancer. Additionally, these aquatic enthusiasts should be advised of the benefits of sun protection strategies such as chemical and physical barriers to reduce the likelihood of developing skin cancer.

## Introduction

Surfing and swimming are two popular recreational aquatic activities in Australia, with an estimated 2.7 million surfers (Stark, 2013) and three million swimmers nationwide (SportAus Ausplay, 2019). These activities are associated with intermittent exposure to ultraviolet radiation (UVR), which is recognized as a causal factor in the development of non-melanoma (NMSC) and melanoma skin cancer (MSC) (Jinna & Adams, 2013). Intermittent exposure to UVR has been well documented to lead to the development of actinic keratosis (AK) lesions, non-melanoma basal cell carcinoma (BCC), squamous cell carcinoma (SCC) and melanoma skin cancer (MSC) (Kanavy & Gerstenblith, 2011). Australia is recognized as having the highest incidence of NMSC and MSC in the world (Elflein, 2020).

Australia is recognized as having the second-highest ultraviolet radiation (UVR) in the world (Elflein, 2020). Within Australia, Southeast Queensland has the second highest UVR index with a UVR range of 4–12 over the year and a mean UVR over 12 months of 7.9. A UVR index of three or greater is recognized as requiring sun protection strategies for the prevention of skin cancer (Gies et al., 2004).

Skin Cancer Australia reported approximately one-third of Australians (30.8%) had skin cancer, making it the most common type of cancer in Australia (Cancer Council of Australia, 2020). Of skin cancers, NMSC, particularly BCC was reported to have the highest standardized rate (1,541 per 100,000) followed by SCC (1,035 per 100,000) and MSC (53.5 cases per 100,000) (Lai, Cranwell & Sinclair, 2018; Australian Institute of Health and Welfare, 2020). Melanoma skin cancers were the costliest in Queensland with the highest proportion of total paid Medicare services related to MSC (~30%) compared to the proportion of Australians living in Queensland (Australian Institute of Health and Welfare, 2016).

The prevalence of skin cancer in swimmers is poorly reported in the literature, with only a single study conducted in the Netherlands (Nelemans et al., 1994) and none to date conducted in Australia. The paucity of research in swimmers is also mirrored when investigating skin cancer in Australian surfers. Climstein et al. (2016) conducted an online survey of 1,348 Australian surfers and reported BCCs as the most prevalent skin cancer (6.8% of all surfers, 9,124 per 100,000) followed by MSC (1.4% of all surfers, 1,854 per 100,000) and SCCs (0.6% of all surfers, 2,670 per 100,000). The MSC rate was more than 34-fold that of the general Australian population (53.5 per 100,000) (Australian Institute of Health and Welfare, 2020). It should be noted that this study used a retrospective design and relied upon the participant’s ability to self-report diagnosed skin cancers. Given the limited evidence on skin cancer in surfers and swimmers, we investigated the point prevalence of NMSC and MSC in surfers and outdoor swimmers in southeast Queensland and Northern New South Wales (latitude −28.016666, longitude 153.399994) through whole-body skin cancer examination.

## Materials and Methods

### Study design

This was a cross-sectional study design incorporating a survey followed by whole-body skin cancer screening. Participants were recruited from notices sent to local general practitioners (GPs), surfing and swimming clubs in addition to local media coverage.

### Survey

The survey consisted of four sections which included participants’ physiological demographics (“Introduction”), activity-specific demographics (peak UVR exposure; “Materials and Methods”) (NSW Government: SafeWork, 2020; Gonzaga, 2009) and history of skin cancer (Results). “Discussion” was completed by a clinician following the screening and included Fitzpatrick skin type (Gupta & Sharma, 2019), type(s) and location(s) of skin cancer(s). “Conclusions” detailed histopathology results. All surveys were completed within the clinic waiting room. If participants had any questions pertaining to the survey, they were answered by either one of the researchers who was onsite or the specialist, depending upon the nature of the query.

### Screening strategy

Whole body skin checks were conducted using a handheld dermatoscope, completed by an accredited skin cancer doctor focusing upon AK, NMSC and MSC using the chaos and clues algorithm (Rosendahl et al., 2012) and prediction without pigment, a decision algorithm for non-pigmented skin malignancy (Rosendahl et al., 2014). These methods have been shown to significantly increase the diagnostic accuracy of detecting AK, NMSC and MSC when used by experienced clinicians (Dinnes et al., 2018; Dinnes et al., 2018). A dermatoscope with 10× magnification and LEDHQ illumination (Heine Delta 30; Heine, Optotechnic GhbH, Herrsching, Germany) was used to inspect all suspect skin lesions. Confirmation of all skin cancers was attained *via* histopathology from a commercial laboratory.

### Statistical analysis

Normality of data was assessed *via* kurtosis, skewness, Q–Q plots and the Kolmogorov–Smirnov test (with Lilliefors significance correction). Heteroscedasticity was assessed with Levene’s inferential test. Statistical analyses were completed using IBM’s Statistical Package for Social Sciences (SPSS, Ver. 27.0), Excel (Microsoft Office 365; Microsoft Corporation, Redmond, WA, USA) and included demographics, independent sample *t*-tests, Chi-square tests and ANOVA (Bonferroni post-hoc test) were used to determine significance between the number of skin cancers and decade of age groups. A bivariate (two-tailed) Pearson correlation coefficient was used to determine relationships between selected outcome variables (number of skin cancers to age and body mass index). Alpha was set *a priori* at *P* < 0.05 to determine significance between groups.

Point prevalence was determined by the number of surfers or swimmers with an AK, BCC, SCC, SCC *in situ* or MSC divided by the total number in that group (National Institute of Mental Health, 2022). The standardized rate per 100,000 was calculated as, for example, the number of surfers or swimmers identified with a particular skin cancer multiplied by 100,000, then divided by the total number of surfers or swimmers, respectively (Statistics Canada, 2017). Odds ratios (OR) is a ratio of two sets of odds, the odds of the event occurring in one group (*i.e*. surfers) *vs*. the odds of the event occurring in a different group (*i.e*. swimmers). The OR of surfers to swimmers was then determined by the odds in surfers divided by the odds in swimmers (Tenny & Hoffman, 2022). MedCalc statistical software (https://www.medcalc.org/calc/odds_ratio.php) was used to calculate all odds ratios. Comparative standardized rates for AK, BCC, SCC and melanoma for the general Australia population were identified from the literature (Lai, Cranwell & Sinclair, 2018; Australian Institute of Health and Welfare, 2020; Smith, 2019).

#### Ethics approval and informed consent

This study was approved by the Southern Cross University Human Research Ethics committee (11 May, 2020/047). All participants provided signed informed consent prior to participating in this study.

## Results

### Sample characteristics/demographics

A total of 171 participants (males *n* = 94, females *n* = 77; surfers *n* = 116, swimmers *n* = 55) competed the survey and underwent a total body skin check for AK, BCC, SCC *in situ*, SCC, and MSCs. Swimmers and surfers had similar mean age, height, mass, body mass index (BMI) and body surface area (BSA). When groups were combined, there was a significant correlation between the total number of skin cancers identified during the screening and age (*r* = 0.51, *P* < 0.001) and BMI (*r* = 0.373, *P* < 0.001).

The majority of surfers self-rated their surfing skill level as intermediate (59.2%) followed by advanced (36.7%), with the remainder self-classified as beginners (4.2%). All swimmers reported being recreational, whereas the majority of surfers were recreational (89.2%) followed by competing with their local board riding club (5.8%). The majority of surfers reported primarily riding a short board (56.7%), followed by a long-board (20.8%). Swimmers were more experienced (+13.1% years); however, surfers had greater UVR exposure (+34.9%, *P* = 0.172) as estimated *via* activity reported for the previous 12 months (total hours/year). Most surfers (93.3%) surfed year-round, with 100% of participants surfing during the summer months. There were fewer swimmers (65.7%) who swam year-round; however, all swimmers also swam during the summer months.

Both groups reported completing some activity during peak UVR (surfers 70.2% *vs*. swimmer 39.2%, *P* = 0.553). However, both groups were similar in their estimated percentage of selected aquatic activity completed during peak UV (surfers 42.8% *vs*. swimmers 45.8%, *P* = 0.553) (Table 1).

**Table 1:** Participant’s demographics, values are mean/median (± SD), number or percent, 95% CI. Specific *P* value included in parentheses where significant differences existed between groups.

Parameter	Group (*n* = 171)	Surfers (*n* = 116)	Swimmers (*n* = 55)
Age (years)	43.9/43.0 (14.8)	42.7/42.0 (14.1) [40.1–45.4]	46.3/48.0 (15.9) [13.2–66.3]
Weight (kg)	74.7/75.0 (14.3)	75.7/77.0 (14.3) [73.1–78.4]	72.8/71.0 (14.3) [41.2–107.3]
BMI (kg/m^2^) • underweight (n) • normal (n) • overweight (n) • obese (n) unreported	24.5/23.9 (3.4) 1 104 50 15 1	24.6/24.0 (3.4) [24.0–25.3] 1 68 37 9 1	24.3/23.4 (3.6) [16.9–31.5] 0 36 13 6 0
BSA (m^2^)	1.89/1.9 (0.2)	1.91/1.9 (0.2) [1.9–2.0]	1.86/1.8 (0.2) [1.8–1.9]
Experience (years) Aquatic activity • hours/week • weeks/year • total/year • lifetime aquatic hours	26.4/25.0 (17.0) 6.84/6.0 (4.9) 42.8/50.0 (11.6) 305.5/225 (256) 8,311/4,800 (9,961)	25.6/22.0 (16.3) [22.6–28.6] 7.30/6.0 (4.5) [6.5-8.1] 44.1/50 (10.9) [42.1–46.3] 333.5/290 (240) [289–377] 9,134/1,060 (10,323) [7,235–11,033]	28.0/25.0 (18.2) [23.1–32.9] 5.86/4.0 (5.7) [4.3–7.4] 39.9 (12.6)*/45 (*P* = 0.026) [36.5-43.3] 246.5 (281)*/160 (*P* = 0.038) [170–322] 6,575/1,000 (8,993) [4,144–9,006]
UVR • activity during peak UV (yes, %) • Activity percentage during peak UV (%)	88.2 43.5 (27.2)	90.5 42.8/30 (26.2) [37.8–47.9]	83.3 45.1/40 (29.8) [36.1–54.0]
Fitzpatrick Skin Type • Skin type 1 • Skin type 2 • Skin type 3 • Skin type 4 • Skin type 5 • Skin type 6	0 9 34 101 27 0	0 8 25 67 16 0	0 1 9 34 11 0

**Note:**

Where* = *P* < 0.05; BMI = body mass index; BSA = body surface areas.

### Prevention and screening practices

There was no difference with regard to the number of prevention strategies reported between surfers and swimmers (*P* = 0.380, 3.2 *vs*. 3.1, respectively). However, a significantly higher percentage of surfers reported using either a chemical and/or physical prevention strategy as compared to swimmers (100% *vs*. 92.7%, respectively; *P* = 0.003). With regard to scalp protection, a higher percentage of swimmers utilized a swim cap as opposed to surfers wearing a surf hat (43.6% *vs*. 20.7%, respectively; *P* = 0.002). Significantly more surfers utilised a rashie as opposed to swimmers (80.2% *vs*. 49.1%, respectively; *P* < 0.001); however, there were no differences between groups with regards to sunscreen, zinc, and lip balm use, or the reapplication of sunscreen (surfers 44.0% *vs*. swimmers 47.9%).

The majority of each group (surfers 63.8% *vs*. swimmers 72.2%) placed emphasis upon whom conducted the skin examinations, with most (surfers 43.9% *vs*. swimmers 54.2%) choosing to see a skin cancer doctor, followed by their GP (Table 2). The majority of participants (surfers 97.4% *vs*. swimmers 100%) were self-referred for their skin cancer screening in this study, with less than one-half of the participants in each group (surfers, 43.3%; swimmers 40.7%) having undergone a skin cancer check within the previous 12 months. There was also no difference identified between groups with regard to completing regular (self and/or partner) exams for suspicious lesions (surfers 36.2% *vs*. swimmers 35.2%; *P* = 0.897).

**Table 2:** Participant’s prevention and screening demographics (vales are percent). Significance (*) between groups identified in parentheses.

Parameter	Group (*n* = 171)	Surfers (*n* = 116)	Swimmers (*n* = 55)
**Prevention and Screening demographics**			
Uses any prevention strategy (yes, %)	–	100.0	92.7* (*P* = 0.003)
Uses hat • surf hat or swim cap (%)	–	20.7	32.7* (*P* = 0.002)
Uses rashie or wetsuit • yes (%)	–	80.2	49.1 (*P* < 0.001)
Previously underwent skin check • <6 months (%) • 1 year ago (%) • 2 years ago (%) • 3 years ago (%) • 4 years ago (%) • 5 years ago (%) • 5+ years ago (%)	27.1 15.3 5.3 1.2 3.5 7.1 15.3	27.6 15.5 5.2 0.9 3.4 6.9 12.9	25.9 14.8 5.6 1.9 3.7 7.4 20.4
Who performed last skin check (%) • GP • Skin cancer doctor • Dermatologist • Plastic surgeon	36.4 51.0 10.5 2.1	39.0 47.0 12.0 2.0	30.2 60.5 7.0 2.3

### History and risk related to skin cancer

Both groups identified as having a history of skin cancer (surfers 41.4% *vs*. swimmers 36.4%, respectively) and a family history of skin cancer (surfers 52.6% *vs*. swimmers 43.6%). The majority of surfers and swimmers (74.1% *vs*. 69.0%) that experienced blistering sunburns as a child were significantly (*P* < 0.001) more likely found to have at least one skin cancer (BCC, SCC, SCC *in situ*, MSC) identified in this study than those who did not experience blistering sunburns as children. Surfers reported a significantly higher (*P* = 0.025) number of lesions of concern (37.1%) as compared to swimmers (20.0%) prior to being screened (Table 3).

**Table 3:** Participant’s history related to skin cancer (vales are number (*n*) or percent). Significance (*) between groups identified in parentheses.

Parameter	Group (*n* = 171)	Surfers (*n* = 116)	Swimmers (*n* = 55)
Personal history of skin cancer (yes, %)	39.8	41.3	36.4
Family history of skin cancer (yes, %)	49.7	52.6	43.6
History of blistering sunburns as a child (yes, %)	62.0	62.1	61.8
Number of sunburns in previous 12 months (*n*)	589	248	341* (*P* = 0.039)
Any lesions of concern (yes, %)	31.6	37.1	20.0* (*P* = 0.025)
Personal history of skin cancer and skin cancer during screening (yes, %)	29.2	31.9	23.6
Skin cancer identified during screening (yes, %)	42.7	50.0	27.3* (*P* = 0.005)

**Figure 1:**
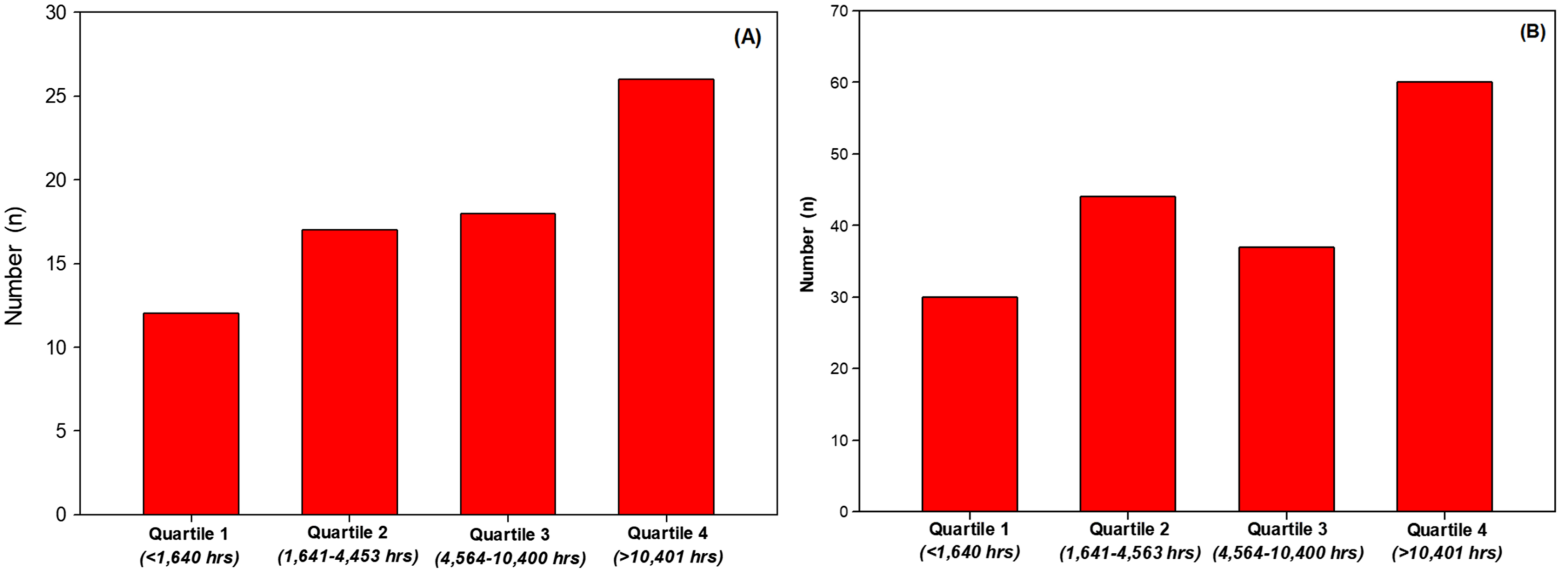
Number of participants identified with a skin cancer per quartile (A) and number of skin cancer identified per quartile (B).

There was no difference (*P* = 0.381) in years’ experience (years surfing or years swimming) or in the number of hours of aquatic activity per week (*P* = 0.075) between groups. With regard to weeks of aquatic activity reported per year, surfers were significantly (+10.5%, *P* = 0.026) more active than swimmers. Surfers over the year spent significantly (+35.3%, *P* = 0.038) more time (total hours per year) in their aquatic activity than swimmers. When lifetime aquatic activity was estimated (number of years’ experience × hours per year), there was no difference (*P* = 0.117) between groups, although surfers, with fewer years’ experience, completed more lifetime aquatic hours than swimmers (+38.9%). However, when groups were combined and aquatic activity expressed in quartiles, there were more participants identified with skin cancers when investigating total aquatic activity exposure (Fig. 1A, quartile 1 *vs*. quartile 4). Likewise, with groups combined, there were more skin cancers identified with increased activity exposure (Fig. 1B, quartile 1 *vs*. quartile 4).

### Skin cancer screening results

The skin specialist identified a total of 74 participants with precancerous AKs during the screening (surfers 59, swimmers 15) and 110 skin cancers (BCC, SCC, SCC *in situ*, MSC). A significantly (*P* < 0.005) greater number of surfers were identified with a skin lesion (PSC, NMSC, MSC) during the screening than swimmers (132 *vs*. 52, respectively; OR 1.85, 95% CI [1.0–3.5]). Surfers also had a higher number (*P* = 0.076) of AK and rate (per 100,000) compared to swimmers (37,068 *vs*. 21,818 respectively, OR 1.7). Surfers also had a higher number of BCCs compared to swimmers (32 *vs*. 10, respectively); however, a lower rate (11,206 *vs*. 14,545 respectively, OR 0.77). Surfers had a slightly higher number of SCC *in situ* than swimmers (24 *vs*. 22; 13,793 *vs*. 12,727, OR 1.08). Surfers had a significantly (*P* = 0.05) higher number of SCCs however, a lower rate of SCCs compared to swimmers (1,724 *vs*. 3,636, OR 3.51). With regard to malignant melanomas, surfers had a higher rate (*P* = 0.137) as compared to the swimmers (5,172 *vs*. 1,818, OR 3.24).

When compared to the general Australian population, surfers and swimmers had higher ORs, which included BCCs (OR 7.27 *vs*. 9.44, respectively), SCCs (OR 1.67 *vs*. 3.51, respectively) and MSC (OR 96.7 *vs*. 34.0, respectively) (Table 4).

**Table 4:** Skin cancer type by group. Values are percent (%), number (*n*) or (95% confidence interval).

Parameter	Surfers: Point prevalence (%)	Swimmers: Point prevalence (%)	Surfers: Standardized rate per 100,000	Swimmers: Standardized rate per 100,000	Odds Ratios (surfers to swimmers)	Comparative standardized rate (per 100,000, or %) in Australia general population	Comparison of standardized rate of Surfers compared to Australia general population standardized rate	Comparison of standardized rate of Swimmers compared to Australia general population standardized rate
Actinic keratosis	37.1	21.8	37,068 [28,826–46,141]	21,818 [12,946–34,370]	2.11 [1.00–4.43]	37–55%^21^	–	–
BCC	11.2	14.5	11,206 [6,667–18,233]	14,545 [7,559–26,160]	0.74 [0.29–1.9]	1,541^8^	7.27	9.44
SCC *in situ*	13.8	30.9	13,793 [8,672–21,236]	12,727 [6,304–24,017]	1.09 [0.42–2.84]	–	–	–
SCC	1.7	3.6	1,724 [474–6,069]	3,636 [321–9,605]	0.94 [0.08–10.7]	1,035^8^	1.67	3.51
Melanoma	5.2	1.8	5,172 [2,392–10,827]	1,818 [322–9,605]	3.24 [0.38–27.6]	53.5^9^	96.7	18.8

**Note:**

Where BCC = basal cell carcinoma; SCC = squamous cell carcinoma; 8 = Lai, Cranwell & Sinclair (2018); 9 = Australian Institute of Health and Welfare (2020); 21 = Tenny & Hoffman (2022).

A MANCOVA demonstrated statistically significant differences in skin cancer totals (total number of skin cancers, total AKs, BCCs, SCC, SCC *in situ*, melanoma) based upon the history of skin cancer (*F* = 3.98, Pillai’s Trace = 1.40, *P* < 0.001) with history of skin cancer as a fixed factor and covariates of Fitzpatrick skin type and age. Adjusting the mean number of skin cancers observed for covariates (age and Fitzpatrick skin type), totals were higher for those with a history of skin cancer (mean with skin cancer history 7.24 *vs*. 3.95 with no skin cancer history, *P* = 0.012). A similar trend was observed for AKs (6.03 *vs*. 3.69, not significant (NS)), total BCC (0.66 *vs*. 0.06, *P* = 0.001), total SCC *in situ* (0.50 *vs*. 0.101, *P* = 0.042), total melanoma (0.62 *vs*. 0.44, NS) and total SCC (0.04 *vs*. −0.01, *P* = 0.024, with the negative mean value due to adjustment to the mean by covariates).

A total of 110 samples were sent for histopathology, and all (100%) samples were confirmed positive as either NMSC (BCC, SCC, SCC *in situ*) or MSC.

### Fitzpatrick skin type and skin cancer

With regard to Fitzpatrick skin type and the number of skin cancers (*P* = 0.013), the majority of skin cancers (groups combined) were seen in the Fitzpatrick skin type 4 (59.1%), this was followed by Type 3 (19.9%) and Type 5 (15.8%) (Table 5). We did not have any participants with a Fitzpatrick type 1 or type 6 skin type. Surfers and swimmers presented with predominantly skin type 4 (57.8% *vs*. 61.8%, respectively), and this was followed by skin type 3 (21.6%) and skin type 5 (13.4%) in surfers and skin type 5 (20.0%) and skin type 3 (16.4%) in swimmers (Table 5).

**Table 5:** Fitzpatrick skin type and number of skin cancers identified during screening.

Number of skin cancers	Skin type 2	Skin type 3	Skin type 4	Skin type 5	Total
0	5	20	51	22	98
1	0	8	24	1	33
2	0	4	11	1	16
3	0	0	7	2	9
4	1	0	3	0	4
5	2	0	3	0	5
6	1	2	0	1	4
7	0	0	0	0	0
8	0	0	1	0	1
9	0	0	1	0	1
Total • Surfers • Swimmers	9 8 1	34 25 9	101 67 34	27 16 11	171 116 55

### Gender differences

There was no significant difference between genders with regard to experience between genders (*P* = 0.253); however, males had significantly more aquatic activity (weeks per year) (*P* = 0.036, +10.6%) than female participants. Females spent significantly more hours (*P* = 0.004, +57.2%) in their activity in peak UVR than male participants. There was no difference regarding the use of any prevention strategies between genders (males 96.8%, females 98.7%). More males reported experiencing sunburns as a child (59.7% *vs*. 63.8%), and a higher percentage of males reported having a sunburn in the previous 12 months (75.5% *vs*. 62.3%). A significantly (*P* < 0.001) higher number of females reported using a tanning bed (31.2% *vs*. 8.5%). More males reported a family history of skin cancer (*P* = 0.084, 44.7% *vs*. 33.8%). Males also had a significantly higher number of skin cancers (all lesions) as opposed to female participants (*P* = 0.011) and a significantly (*P* = 0.042) higher number of NMSC and MCS (combined).

## Discussion

To date, this research represents the largest sample skin cancer screening study conducted by a clinician in surfers and swimmers. This study also contributes to the lack of findings on skin cancer in surfers and swimmers. Surfers had higher standardized rates (per 100,000) of AK, SCC *in situ* and MSC as compared to swimmers, whilst swimmers had a higher standardized rate of BCC and SCC. Both aquatic groups (individually and combined) had rates higher than the general Australian population for NMSC and MSC (Lai, Cranwell & Sinclair, 2018; Australian Institute of Health and Welfare, 2020). Given the increased UVR exposure (both direct and reflective from the water), this is not a surprising finding. However, the consistently higher comparative rates of NMSC and MSC are a concerning finding.

The point prevalence rate of PSC (surfers 37.1%, swimmers 21.8%) is within the estimated percent reported in the general Australian population for surfers (37–55%) (Smith, 2019); however, swimmers had a lower point prevalence rate. It should be noted that these precancerous AK lesions are well recognized as being precursors to the development of BCCs and SCCs (Smith, 2019). Therefore, it is reasonable that participants identified with AKs in this study will likely have lesions that will develop into BCC or SCC lesions sometime in the foreseeable future. Dodds, Chia & Shumack (2014) reported the progression of AKs to invasive SCCs was approximately one to 10% over 10 years. Ratushny et al. (2012) reported that individuals with multiple AKs have up to an 80% risk of developing invasive SCCs and with the risk increasing for those individuals with five or more AKs. In our study, we had 52 participants (40 surfers, 12 swimmers) identified with five or more AKs. Brougham et al. (2012) conducted a retrospective study of New Zealanders and reported that approximately 2.5% of SCCs metastasized, with approximately 90% reaching lymph nodes.

The standardized rates (per 100,000) of BCC, SCC and MSC were consistently higher than the standardized rates (BCC surfers 27,568, swimmers 18,181 *vs*. 11.8; SCC surfers 9,482, swimmers 7,272 *vs*. 1,035; melanoma surfers 5,172, swimmers 1,818 *vs*. 53.5) previously reported in the general Australian population. More recently, the Queensland Cancer Statistics registry (Cancer Council Queensland, 2018), reported the melanoma rate at 77.0 per 100,000 (95% CI [74.7–79.4]) which is well below our reported rate for melanoma. The rates we reported in this study are similar to rates (per 100,000) previously reported in Australian surfers by Climstein et al. (2016) (BCC 9,124; SCC 2,670; melanoma 1,854). However, the previous study mythologies by Climstein et al. (2016) incorporated an online survey as opposed to screening by a clinician.

Iannacone et al. (2015) previously investigated MSC in Australian adolescents and young adults based upon de-identified data from the Queensland Cancer Registry. They reported an annual incidence of 10.1 per 100,000 for invasive MSC. Our equivalent MSC rate was 180 to 512-fold comparatively. Unfortunately, the study by Iannacone et al. (2015) focused upon tumour type and did not contain exposure metrics related to UVR. Climstein et al. (2016) reported a rate of 1,854 per 100,000, which is near identical to the rate found in this study in swimmers (1,818 per 100,000); however, it is below the rate we identified in surfers in this study (5,172 per 100,000). Although Climstein et al. (2016) did not report experience in their previous study, the surfers in this current study were approximately 20% older, with the exposure (expressed in hours surfing per year) similar to that reported in the current study (mean 305 *vs*. 330 h/year). The actual skin cancer rate is may have been under reported in the survey study (Climstein et al., 2016) as it relied upon participants self-reporting as opposed to the objective methods used in the current study. It is feasible the higher rate of MSC found in the surfers in the present study is attributed to a greater exposure over the lifetime and the methodology of clinical screening as opposed to a survey.

Moehrle (2008) previously reported that beach and water sports (and sunburn) are independent risk factors for the BCC development; it is reasonable that surfing and swimming were, therefore, an independent risk factor for the increased rates seen in NMSCs and MSCs in this study. Both groups reported a similar history of sunburns (surfers 62.1%, swimmers 61.8%); however, swimmers reported a significantly (*P* = 0.03) higher number of sunburns (*n* = 446) as compared to surfers (*n* = 353). Sunburn in surfers has previously been reported whereas De Castro-Maqueda et al. (2020) reported the majority (76.7%) of surfers in their study reported experiencing three or more sunburns in the previous season. These same authors also described in a separate study (De Castro-Maqueda et al., 2021) that approximately one-half of their participants used inadequate sun protection. Regrettably, we did not inquire into the severity nor duration of sunburns in participants in our current study. Additionally, AK, BCC and SCC have been shown to be associated with an increased risk (4.3-fold) of developing a MSC (Wernli et al., 2016), thereby increasing risk of MSC in our participants who, when screened, were clear of MSC. Therefore, these participants who were not identified with an MSC in the present study are at risk of developing MSC in the future.

Solar UVR exposure and resultant skin cancers are dependent upon a number of geographical, behavioural and genetic susceptibility factors. Ultra-violet radiation has been estimated to cause approximately 95% of MSCs in areas of high exposure (Armstrong & Kricker, 1993). Despite the high usage of chemical and/or physical protection strategies reported in our participants, our participants had a high point prevalence of NMSC and MSCs, suggestive of the long-term, detrimental effects of UVR exposure whilst surfing or swimming. Furthermore, there is a direct correlation between sun exposure and UVR exposure, which accounts for 95% of skin cancer cases. This risk is maintained in those participants continuing to surf or swim, which is highly likely as anecdotally, no participants commented upon ceasing their aquatic activity based upon a PSC, NMSC or MSC identified during their skin cancer screening.

It is well recognized that Fitzpatrick skin types 1 and 2 are at the highest risk of developing skin cancer due to the reduced pigmentation which affords a natural protection from UVR exposure. In our study however, we found the greatest prevalence of skin cancer was in Fitzpatrick type 4 followed by type 3. Despite the widespread use of Fitzpatrick skin typing, it has been rarely applied in similar research. For example, Climstein et al. (2016) assessed skin type, however, the Fitzpatrick skin type was simplified to only fair to black, contrary to its intended clinical application. Other related studies (Dozier et al., 1997; Lawler et al., 2007; Zalaudek et al., 2020; Noble-Jerks, Weatherby & Meir, 2006) neglected to investigate Fitzpatrick skin type altogether, therefore limiting comparisons to our findings related to skin type.

### Strengths and Limitations

This was the first study in Australia assessing the point prevalence of PSC, NMSC and MSC in surfers and swimmers *via* total body skin checks conducted by a clinician. Further strengths include our methodology included screening conducted by a specialist as opposed to participant recall *via* a survey. Additionally, the confirmation of all (100%) histopathology samples by commercial laboratory analysis matched the clinician’s findings.

Our participants had a high degree of homogeneity as they were from a limited geographic locale; however, this restrictive inclusion criteria limits the ability to extrapolate our findings to other surfers and swimmers within Australia, or elsewhere in the world. However, intermittent exposure to UVR is well documented in the literature as a casual mechanism for the development of NMSC and MSC, and we believe our findings have wide, geographic relevance.

It has, however, been reported that Fitzpatrick’s skin type is a confounding variable for the development of NSMC and MSC (Ting et al., 2007). Although Fitzpatrick’s skin type was assessed by a clinician, we cannot determine the effect Fitzpatrick skin type had on the development of NMSC and MSC in our participants, rather recognize that skin type is recognized as contributory to the development of skin cancer. Additionally, we did not note ethnicity, which may account for the skew in the skin types of participants in our study. Additionally, a significant number of the participants were very tanned with significant sun damage, making it difficult to assess Fitzpatrick skin type.

A limitation of the present study was that the sample size was relatively small. However, when compared to the very limited number of skin cancer screening studies that incorporated clinician screening (Dozier et al., 1997; Douglas, Baillie & Soyer, 2011), we did exceed the participant numbers in those previously published similar studies. We also recognize we did not account for confounding factors such as occupational and other UVR exposure (*i.e*., other outdoor activities), which would have contributed to the point prevalence’s reported in this study. As it was not possible to attain a list or number of surfers in the area, the participants were self-selected. Therefore, we were not able to utilize random sampling, and as such, selection bias may have occurred. As participants were self-referred to the study, it is possible we attracted participants who were more concerned about their health with regard to skin cancer, and this would represent an additional bias. Additionally, as the total number of surfers and swimmers in the region is unknown, we cannot assess nor calculate a response rate, and therefore determine the representativeness of our participants to their respective aquatic groups. We believe there is no confounding bias as we did not investigate casual relationships.

## Conclusions

The literature regarding point prevalence of skin cancer in Australian swimmers (and elsewhere in the world) is completely undocumented, and the point prevalence determined *via* screening in Australian surfers is poorly documented given the worldwide popularity of surfing. The point prevalence for NMSC and MSC in our participants who surf and swim was much higher than the comparative general Australian population. This study adds insight and heightens the awareness of clinicians to screen their patients who participate in surfing and swimming for the early detection and treatment of pre-invasive or early-stage invasive MSCs for improved health outcomes and reduced mortality (Iannacone et al., 2015). Patients should also be educated on the benefits of sun protection behaviour strategies such as chemical and physical barriers as they have been previously shown to be effective (Moehrle, 2008; Sander et al., 2020). Further research into other aquatic enthusiasts such as stand up paddle boarders and kayakers and outdoor enthusiasts such as walkers, joggers and cyclists are warranted.

## Supplemental Information

10.7717/peerj.13243/supp-1 Supplemental Information 1 The type of pre-cancerous, non-melanoma and melanoma skin cancer per group and location on the body.

10.7717/peerj.13243/supp-2 Supplemental Information 2 The type of demographics of years’ experience, and aquatic activity (hours per week, weeks per year and total hours per year).

10.7717/peerj.13243/supp-3 Supplemental Information 3 The outcome variables from the study.

10.7717/peerj.13243/supp-4 Supplemental Information 4 Participant information sheet.

10.7717/peerj.13243/supp-5 Supplemental Information 5 Research Survey.

## Additional Information and Declarations

### Data Availability

The following information was supplied regarding data availability: The raw data is available in the Supplemental Files.
