# Supplementary material for: Point prevalence of non-melanoma and melanoma skin cancers in Australian surfers and swimmers in Southeast Queensland and Northern New South Wales
Source: PeerJ. 2022 Apr 28;10:e13243. doi: 10.7717/peerj.13243 (PMC9057286; doi:10.7717/peerj.13243)
Supplement: Supplemental Information 4 [file peerj-10-13243-s004.docx]

**PARTICIPANT INFORMATION SHEET**

**Prevalence, Types and Treatments of Skin Cancer in Surfers and Swimmers**

Southern Cross University

School of Health and Human Sciences

Gold Coast Campus

Dear Sir/Madam,

Thank you for expressing an interest in participating in the research project “Prevalence, Types and Treatments of Skin Cancer in Surfers and Swimmers”.

Attached is an Information Sheet about this project. Please read it carefully and if you decide to participate, please complete and sign the Consent Form to and return it to me (b.doyle.16@student.scu.edu.au) either by scanning it or via post:

Brendan Doyle

c/o Dr Mike Climstein

Clinical Exercise Physiology

School of Health and Human Sciences

B7.39 – Gold Coast

BILINGA, QLD 4225

Alternatively, if you have any questions about the research project, please call us on 07 5589 3330 and we can answer any questions you may have.

Yours sincerely,

Brendan Doyle, Honours Candidate

c/o Dr Mike Climstein (FASMF, FACSM, FAAESS)

Clinical Exercise Physiology

School of Health and Human Sciences

B7.39 – Gold Coast

T 07 5589 3330

E: [b.doyle.16@student.scu.edu.au](mailto:b.doyle.16@student.scu.edu.au)

**PARTICIPANT INFORMATION SHEET:**

*This information sheet is yours to keep*

**Prevalence, Types and Treatments of Skin Cancer in Surfers and Swimmers**

The research team members responsible for conducting this research are identified at the end of this information sheet, along with their contact details.

**Brief background to project:** Surfing and swimming are two of the most popular outdoor aquatic activities in Australia with an estimated 2.7 million recreational surfers and 1.5 million recreational swimmers. As a result, the expected risk of skin cancer in surfers and swimmers due to long periods of exposure to ultraviolet radiation, wearing less clothing and reflection from water is of great concern. According to the Australian Cancer Council, nearly one-third of all Australians have had skin cancer at some time in their lives, making it the most common type of cancer in Australians. Therefore, the purpose of this study is to evaluate the prevalence and types of skin cancer identified in participants who attend a skin cancer clinic.

**What this project involves:** The following describes the nature of your involvement in this research, should you agree to participate:

- Answering questions regarding your demographics (age, height, weight, ethnicity).
- Answering questions regarding your surfing, swimming demographics (hours per week, weeks per year, do you surf, swim or SUP during peak ultraviolet radiation periods). We will also inquire about any prevention strategies you commonly use such as sunscreen/zinc use, lip balm, hat/swim cap and rashie.
- It is anticipated completion of this survey may take a minimum of 10 minutes.
- Following completion of the survey the Specialist assess your skin colour and skin type and then completes a comprehensive skin check to identify any skin cancers or suspicious moles. If you have any skin cancers identified, the type (basal cell carcinoma or squamous cell carcinoma or melanoma) will be noted. If a skin cancer has been identified, the treatment utilized will be noted.

**Inclusion criteria:** You need to meet the following criteria to be eligible to participate:

- All participants aged 18 and older and swim or surf
- Provide written, informed consent

**Exclusion criteria:** if you have any of the following criteria you are unfortunately not eligible to participate:

- Unwilling to provide written informed consent

**Possible discomforts and risks:**

There are minimal risks or discomforts associated with participation in this research project. If you become distressed whilst completing the survey, please stop immediately and withdraw from the research project. If you feel distress, please contact Beyond Blue on 1300 22 4636.

**Your role:**

You are free to withdraw from this research project at any time with no penalty or prejudice. You do not have to explain why you wanted to withdraw. If you consent to participate in this research, please complete and sign the Consent Form and return it to the receptionist before you see the Specialist.

**Outcomes:**

The outcomes of this research will be published in a peer-reviewed journal and presented at national or international conferences, but only group data will be reported. It is expected that the outcomes of this research will better inform surfers and swimmers on the lifetime prevalence of skin cancer and prevention strategies.

**Feedback:**

If you would like to receive a summary of the project results when it is finished, tick “Yes- I would like to receive feedback on this study” on the Informed Consent form.

Should you have any questions about the above please do not hesitate to contact any of the following members of the research team:

| 1. Dr Mike Climstein   T: (07) 5589 3330  E: [Michael.climstein@scu.edu.au](mailto:Michael.climstein@scu.edu.au) | A. Brendan Doyle  T: (07) 5601 0495  E: [b.doyle.16@student.scu.edu.au](mailto:b.doyle.16@student.scu.edu.au) |
| --- | --- |

| *This project has been approved by the Human Research Ethics Committee of Southern Cross University; approval number 2020/047. If you have concerns about the* ***ethical conduct*** *of this research or the researchers, the following procedure should occur. Write to the following:* | | |
| --- | --- | --- |
| *The Ethics Complaints Officer* | *Southern Cross University PO Box 157 Lismore NSW 2480* | *Email: ethics.lismore@scu.edu.au* |

*All information is confidential and will be handled as soon as possible if you consent to participate in this research.*
