# Supplementary material for: Point prevalence of non-melanoma and melanoma skin cancers in Australian surfers and swimmers in Southeast Queensland and Northern New South Wales
Source: PeerJ. 2022 Apr 28;10:e13243. doi: 10.7717/peerj.13243 (PMC9057286; doi:10.7717/peerj.13243)
Supplement: Supplemental Information 5 [file peerj-10-13243-s005.docx]

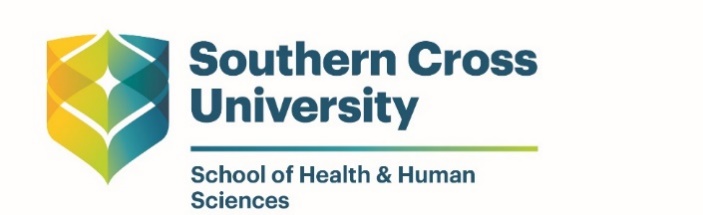

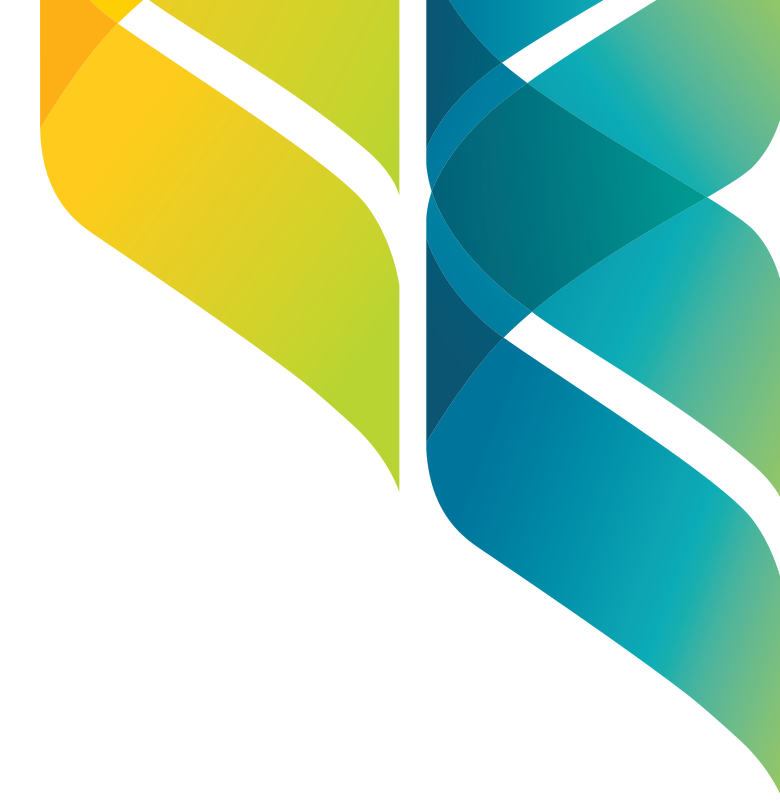


**RESEARCH SURVEY**

**Prevalence, Types and Treatment of Skin Cancer in Surfers, Swimmers and Stand Up Paddle Boarders**

**Participants are asked NOT TO put their name or any other identifying information on the survey**

**Clinic ID: ___________ ꙱ Self-referred ꙱ Referred by your GP**

**Age: _______ Height (cm): _______ Weight (kg): _______**

**Gender:** ꙱ Male ꙱ Female

**Smoking status:** ꙱ Smoker ꙱ Non-smoker ꙱ Ex-smoker

꙱ Stand-Up Paddle Boarding

**PRIMARY recreational activity:** ꙱ Surfing ꙱ Swimming

**Please fill out your PRIMARY recreational activity only.**

**SURFING (if main recreational activity or sport)**

**Surfing experience (years):**

**Surfing hours per week: Surfing weeks per year:**

**Surfing ability**: ꙱ Beginner ꙱ Intermediate ꙱ Advanced

**Surfing status**: ꙱ Recreational ꙱ Competitive (local board rider) ꙱ Competitive (pro)

**Board type (primary):** ꙱ Short board ꙱ Mini-mal ꙱ Long-board

**Surf stance:** ꙱ Natural (left leg forward) ꙱ Goofy (right leg forward)

**Surfing season (tick all that may apply):** ꙱ Spring ꙱ Summer ꙱ Autumn ꙱ Winter

**Do you ever surf during peak ultra violet radiation (i.e., sun strongest from 10am to 3pm):**

꙱ Yes ꙱ No Estimated percent of surfing time during peak UV:___________

**SWIMMING (if main recreational activity or sport)**

**Swimming experience (outdoors only, years):**

**Swimming hours per week: Swimming weeks per year:**

**Swimming season (tick all that may apply):** ꙱ Spring ꙱ Summer ꙱ Autumn ꙱ Winter **Do you ever swim during peak ultra violet radiation (i.e., sun strongest from 10am to 3pm):**

꙱ Yes ꙱ No Estimated percent of swimming time during peak UV:___________

**STAND UP PADDLE BOARDING (SUP, if main recreational activity or sport)**

**SUP experience (years):**

**SUP hours per week: SUP weeks per year:**

**SUP ability**: ꙱ Beginner ꙱ Intermediate ꙱ Advanced ꙱ Professional

**SUP stance:** ꙱ Natural (left leg forward) ꙱ Goofy (right leg forward)

**SUP season (tick all that may apply):** ꙱ Spring ꙱ Summer ꙱ Autumn ꙱ Winter

**Do you ever SUP during peak ultra violet radiation (i.e., sun strongest from 10am to 3pm):**

꙱ Yes ꙱ No Estimated percent of SUP time during peak UV:___________

**SKIN CANCER PREVENTION STRATEGIES**

**Which of the following sun protection strategies do you use on a regular basis? (tick all that may apply)**

꙱ Surf hat ꙱ Swim cap

꙱ Short sleeve rash vest (or wetsuit) ꙱ Long sleeve rash vest (or wetsuit)

**Do you wear sunscreen?** ꙱ Yes ꙱ No

꙱ Sunscreen/zinc nose ꙱ Sunscreen/zinc face & neck ꙱ Sunscreen all exposed skin

**If yes, do you reapply sunscreen as directed?** ꙱ Yes ꙱ No

**Do you use SPF lip balm?** ꙱ Yes ꙱ Yes and reapply as directed ꙱ No

**Do you conduct self/partner exams for suspicious moles on a regular basis?**

꙱ Yes ꙱ No

**How long ago was your last skin check for skin cancer or suspicious moles?**

꙱ < 6 months ꙱ 1 year ꙱ 2 years ꙱ 3 years ꙱ 4 years ꙱ 5 years ꙱ 5 years+ ꙱ Never

**Who performed this skin check?**

꙱ Skin cancer doctor ꙱ GP ꙱ Dermatologist ꙱ Plastic Surgeon

**Do you place any significance on who is doing the skin check?**

꙱ Yes ꙱ No

**Fitzpatrick Skin Type (completed with the Doctor)**

**Eye colour:** ꙱ Light blue/grey/green ꙱ Blue/grey/green ꙱ Hazel/light brown

꙱ Dark brown ꙱ Brownish black
**Natural hair colour:** ꙱ Red/light blonde ꙱ Blonde ꙱ Dark blonde or light brown

꙱ Dark brown ꙱ Black
**Natural skin colour:** ꙱ Ivory white ꙱ Fair or pale ꙱ Fair to beige with golden undertone

꙱ Olive or light brown ꙱ Dark brown or black
**Freckles on exposed or unexposed areas:** ꙱ Many ꙱ Several

꙱ A few ꙱ Very few ꙱ None
**How does your skin respond to the sun?:** ꙱ Always burns/blisters/peels ꙱ Often burns/blisters/peels

꙱ Burns moderately ꙱ Burns rarely, if at all ꙱ Never burns
**Does your skin tan?:** ꙱ Never, I always burn ꙱ Rarely

꙱ Sometimes ꙱ Often ꙱ Always
**How deeply do you tan?:** ꙱ Not at all/very little ꙱ Lightly

꙱ Moderately ꙱ Deeply ꙱ Skin naturally dark
**How sensitive is your face to the sun?:** ꙱ Very sensitive ꙱ Sensitive

꙱ Normal ꙱ Resistant ꙱ Very resistant

**Family history of skin cancer?** ꙱ Yes ꙱ No ꙱ Unsure **Blistering sunburns as a child?** ꙱ Yes ꙱ No ꙱ Unsure **Ever used a solarium/tanning bed?** ꙱ Yes ꙱ No **Number of sunburns previous year:**
 ꙱ 0 ꙱ 1 ꙱ 2 ꙱ 3 ꙱ 4 ꙱ 5+
**Do you have any lesions of concern?** ꙱ Yes ꙱ No **Do you have a history of skin cancer?** ꙱ Yes ꙱ No **If yes, tick body part, then tick relevant type of skin cancer (if known) and treatment:**

**Skin Cancer Risk and History**

**꙱ Scalp Type: ꙱** Basal cell ꙱ Squamous cell ꙱ Melanoma ꙱ Unsure **Treatment: ꙱** Burnt off (cream, cold or heat) ꙱ Surgical removal

**꙱ Nose Type: ꙱** Basal cell ꙱ Squamous cell ꙱ Melanoma ꙱ Unsure **Treatment: ꙱** Burnt off (cream, cold or heat) ꙱ Surgical removal

**꙱ Face Type: ꙱** Basal cell ꙱ Squamous cell ꙱ Melanoma ꙱ Unsure **Treatment: ꙱** Burnt off (cream, cold or heat) ꙱ Surgical removal

**꙱ Lip Type: ꙱** Basal cell ꙱ Squamous cell ꙱ Melanoma ꙱ Unsure **Treatment: ꙱** Burnt off (cream, cold or heat) ꙱ Surgical removal

**꙱ Ear Type: ꙱** Basal cell ꙱ Squamous cell ꙱ Melanoma ꙱ Unsure **Treatment: ꙱** Burnt off (cream, cold or heat) ꙱ Surgical removal

**꙱ Neck Type: ꙱** Basal cell ꙱ Squamous cell ꙱ Melanoma ꙱ Unsure **Treatment: ꙱** Burnt off (cream, cold or heat) ꙱ Surgical removal

**꙱ Shoulder Type: ꙱** Basal cell ꙱ Squamous cell ꙱ Melanoma ꙱ Unsure **Treatment: ꙱** Burnt off (cream, cold or heat) ꙱ Surgical removal

**꙱ Chest Type: ꙱** Basal cell ꙱ Squamous cell ꙱ Melanoma ꙱ Unsure **Treatment: ꙱** Burnt off (cream, cold or heat) ꙱ Surgical removal

**꙱ Arm Type: ꙱** Basal cell ꙱ Squamous cell ꙱ Melanoma ꙱ Unsure **Treatment: ꙱** Burnt off (cream, cold or heat) ꙱ Surgical removal

**꙱ Back Type: ꙱** Basal cell ꙱ Squamous cell ꙱ Melanoma ꙱ Unsure **Treatment: ꙱** Burnt off (cream, cold or heat) ꙱ Surgical removal

**꙱ Hand Type: ꙱** Basal cell ꙱ Squamous cell ꙱ Melanoma ꙱ Unsure **Treatment: ꙱** Burnt off (cream, cold or heat) ꙱ Surgical removal

**꙱ Upper leg Type: ꙱** Basal cell ꙱ Squamous cell ꙱ Melanoma ꙱ Unsure **Treatment: ꙱** Burnt off (cream, cold or heat) ꙱ Surgical removal

**꙱ Lower leg Type: ꙱** Basal cell ꙱ Squamous cell ꙱ Melanoma ꙱ Unsure **Treatment: ꙱** Burnt off (cream, cold or heat) ꙱ Surgical removal

**꙱ Foot Type: ꙱** Basal cell ꙱ Squamous cell ꙱ Melanoma ꙱ Unsure **Treatment: ꙱** Burnt off (cream, cold or heat) ꙱ Surgical removal

**꙱ Other: ________________________________**

**Thank you for completing our survey. If you would like a copy of the results of our findings from the study, please ensure you wrote your email address on the informed consent form.
 NOTE: Please do not include your name or any other identifying information on this form.**

**TO BE COMPLETED BY YOUR DOCTOR/SURGEON**

**Actinic/solar keratosis (pre skin Ca) diagnosed:**

**꙱ Top of head ꙱ Face ꙱ Lip ꙱ Nose ꙱ Ear ꙱ Neck ꙱ Shoulder**

**꙱ Chest ꙱ Arm ꙱ Back ꙱ Hand ꙱ Upper leg ꙱ Lower leg**

**꙱ Foot ꙱ Other: ______________________________________________________**

**BCC diagnosed:**

**꙱ Top of head ꙱ Face ꙱ Lip ꙱ Nose ꙱ Ear ꙱ Neck ꙱ Shoulder**

**꙱ Chest ꙱ Arm ꙱ Back ꙱ Hand ꙱ Upper leg ꙱ Lower leg**

**꙱ Foot ꙱ Other: ______________________________________________________**

**SCC (in situ/IEC) diagnosed:**

**꙱ Top of head ꙱ Face ꙱ Lip ꙱ Nose ꙱ Ear ꙱ Neck ꙱ Shoulder**

**꙱ Chest ꙱ Arm ꙱ Back ꙱ Hand ꙱ Upper leg ꙱ Lower leg**

**꙱ Foot ꙱ Other: ______________________________________________________**

**SCC diagnosed:**

**꙱ Top of head ꙱ Face ꙱ Lip ꙱ Nose ꙱ Ear ꙱ Neck ꙱ Shoulder**

**꙱ Chest ꙱ Arm ꙱ Back ꙱ Hand ꙱ Upper leg ꙱ Lower leg**

**꙱ Foot ꙱ Other: ______________________________________________________**

**Melanoma diagnosed:**

**꙱ Top of head ꙱ Face ꙱ Lip ꙱ Nose ꙱ Ear ꙱ Neck ꙱ Shoulder**

**꙱ Chest ꙱ Arm ꙱ Back ꙱ Hand ꙱ Upper leg ꙱ Lower leg**

**꙱ Foot ꙱ Other: ______________________________________________________**

**Treatment required: ꙱ Yes ꙱ No ꙱ No: wait and watch**

**Treatment performed: ꙱ Cryotherapy ꙱ Topical therapy ꙱ Curettage & cautery**

**꙱ Excision + direct closure ꙱ Excision + flap repair ꙱ Excision + graft**

**꙱ Local anaesthetic ꙱ General anaesthetic**

**꙱ Radiotherapy required ꙱ Sentinel node biopsy**

**Pathology Results:**

**Punch Biopsy: ꙱ Confirmed + ꙱ Confirmed -**

**Shave Biopsy: ꙱ Confirmed + ꙱ Confirmed -**

**Excision: ꙱ Confirmed + ꙱ Confirmed -**
